# New Insights into the Pathophysiology and Treatment of Fibromyalgia

**DOI:** 10.3390/biomedicines5020022

**Published:** 2017-05-13

**Authors:** Tobias Schmidt-Wilcke, Martin Diers

**Affiliations:** 1 Department of Neurology, St. Mauritius Therapieklinik, 40670 Meerbusch, Germany; 2 Department of Clinical Neuroscience and Medical Psychology, Medical Faculty, Heinrich-Heine-University, 40225 Düsseldorf, Germany; 3 Department of Psychosomatic Medicine and Psychotherapy, LWL University Hospital, Ruhr-University Bochum, 44791 Bochum, Germany; martin.diers@ruhr-uni-bochum.de

**Keywords:** fibromyalgia, chronic pain, resting state, fMRI (functional magnetic resonance imaging), learning

## Abstract

Fibromyalgia is characterized by chronic widespread pain and several additional symptoms such as fatigue, cognitive dysfunction, depressive episodes, and anxiety. The underlying pathophysiology of fibromyalgia is still poorly understood, and treatment is often unsatisfactory. Current research provides evidence for altered pain processing in chronic pain patients, and specifically in fibromyalgia patients, possibly based on altered functional connectivity and brain chemistry in brain regions within the pain processing system. Besides discussing evidence from studies applying brain imaging (specifically resting state fMRI (Functional magnetic resonance imaging)), the current review aims at providing an overview of pharmacological and non-pharmacological treatment options. We will also summarize the most important results from recently performed brain imaging studies providing new insights into the potential mechanisms of various therapeutic approaches.

## 1. Introduction

Chronic pain is a serious public health problem affecting about 15% of the adult population in Western industrialized countries [1,2,3]. While there have been tremendous advances in the treatment of chronic pain disorders, for a substantial subset of these individuals, pain management remains a significant problem. Emerging data suggests that a common feature associated with treatment failure is pain arising from dysfunctions within the brain and spinal cord [4,5]. A canonical centralized pain disorder is fibromyalgia (FM)—a common chronic pain condition characterized by widespread pain which affects nearly 2%–4% of the general population [6,7]. Other common pain disorders, such as pelvic pain, chronic low back pain, and osteoarthritis of the knee may affect at least 10% of the population [8,9].

Besides chronic widespread pain, FM patients often experience further symptoms, such as fatigue, sleep disturbances, cognitive dysfunction, depressive episodes, and anxiety. Although the underlying pathophysiology is still poorly understood and treatment is often unsatisfactory, the recognition and diagnosis of FM has significantly improved over the last years, and consequently there has been an increase in the availability of therapeutic options for patients. Furthermore, research into the neurobiological and psychological mechanisms that contribute to chronic pain and concomitant symptoms in FM patients has advanced our understanding of this debilitating disorder.

This review aims at summarizing some important findings in the fields of biomedical and psychological research, with a specific focus on brain imaging and neurophysiology in chronic pain and FM.

## 2. Current Pathophysiological Concepts of Chronic Pain and Fibromyalgia

### 2.1. Medical Concepts

Current research tries to identify risk factors and pathophysiological mechanisms that contribute to the development of chronic pain. A number of genetic and behavioral risk factors have already been identified, including certain genetic predispositions [10,11], but also distress in daily life and traumatic events are thought to be associated with the development of chronic pain [12,13]. Apart from genetic and psychological considerations, functional magnetic resonance imaging (fMRI) has substantially advanced the field. A number of brain imaging studies have reported an increased activation of the pain processing network in fibromyalgia patients (as compared to healthy controls) in response to nociceptive stimuli, implying the presence of a hyper-active pain detection and processing system [14,15,16]. Other studies provide evidence that FM patients display reduced activation or connectivity within the pain inhibitory network [17]. Looking at cross-modal sensory hypersensitivity (a phenomenon frequently observed in FM patients), it could also be demonstrated that insular activity evoked by an aversive level of visual stimulation was significantly increased in FM patients as compared to healthy controls and also correlated with pain intensity, suggesting an altered, possibly maladaptive, cross-talk between various sensory modalities [18].

More recently, resting state fMRI has been applied to look at patients at rest, rather than reacting to a painful stimulus, with the intention to possibly better identify markers of spontaneous (clinical) pain. Altered resting state functional connectivity (rs-fc) could be detected between the default mode network (DMN) and the insular cortex (IC) [19], as well as between the mid IC and the midcingulate cortex/medial frontal gyrus [20], with FM patients showing an increased connectivity between these structures. With respect to pain perception, the IC as a central hub within the pain perception network is involved in both the encoding of pain intensity and somatotopy (posterior IC), and also in affective pain processing and learning (anterior IC) [21]. In other words, in FM it seems to be the case that a hyperconnectivity of the IC to other components of the pain processing network [22] and other networks involved in self-awareness and self-monitoring (i.e., the DMN) makes the brain vulnerable to increased pain perception and the development of a chronic pain state.

Another neuroimaging technique is proton magnetic resonance spectroscopy. This noninvasive method can quantify the concentration of various metabolites, among them the two most important neurotransmitters: glutamate and GABA (γ-amino-butyric-acid). Emerging evidence suggests a mismatch of excitatory and inhibitory neurotransmitter concentrations—either increased levels of the excitatory neurotransmitter glutamate/glutamine or decreased levels of the inhibitory neurotransmitter GABA [23]—in the pain processing region in pain patients, specifically the IC. Interestingly these neurochemical alterations also seem to have an effect on rs-fc, which in combination seem to be important factors for both lowered pain thresholds (i.e., hyperalgesia) and the genesis of chronic pain.

### 2.2. Psychological Concepts

Associative learning such as operant or Pavlovian conditioning can influence the processing of pain on all levels—the verbal-subjective, the behavioral, and the physiological [24]. Fordyce [25] proposed that positive as well as negative reinforcement of pain behaviors (such as sighing or grimacing) and a lack of positive reinforcement of healthy behaviors (such as movement or smiling) can increase the expression of pain behaviors and over time lead to behaviorally induced chronic pain problems [25].

Direct verbal reinforcement of pain has been identified as an important modulator of the pain response. When, for example, chronic back pain (CBP) patients and healthy controls were reinforced for increasing or decreasing their verbal pain responses, both patients and controls learned this task equally well; however, the patients showed a delay in the extinction of the verbal pain response. The late event-related response of the somatosensory evoked potential (>250 ms) was unaltered, and showed mainly habituation. However, the early response (N150) was affected by the conditioning procedure and remained high in the CBP group that had been reinforced for higher pain ratings during extinction. This indicates a direct effect of verbal reinforcement on the early cortical processing of nociceptive information [26]. The lack of extinction in cortical processing implies that maladaptive learnt physiological responses may greatly contribute to pain chronicity.

Chronic pain patients might also have learned to increase muscle tension in anticipation of painful stimuli to reduce pain. This would result in negative reinforcement and could lead to short-term pain reduction, but on the long term stimulate and sensitize nociceptors and thus increase pain. During painful stimuli on the lower arm or back, CBP patients were instructed to increase their muscle tension or keep it low. During the tension increase condition, the CBP patients but not the healthy controls (HCs) showed higher N150 and N150/P260 amplitudes [27]. In patients with FM, movements or certain muscle contractions could already be painful. During muscle contractions, it could be found that patients with FM had increased muscle tension compared to HC [28]. Thus, operantly conditioned muscle tension could contribute to chronicity.

In a study in which pain was implicitly reinforced, a series of tonic painful heat stimuli were applied to the dominant hand. The healthy participants had to adjust the temperature at the end of each trial to the subjective temperature felt at the beginning of each trial, which was objectively not changed. The temperature was increased or decreased in each subsequent trial, depending on the adjustment in the trial before. Thus, the behavior of the subjects was reinforced without their knowledge. It was shown that increased or decreased pain sensitivity could be implicitly learned [29]. In another study with healthy participants, sensitization could be modulated by implicit reinforcement [30]. Thus, operant learning mechanisms based on intrinsic reinforcement may provide an explanation for the gradual development of sustained hypersensitivity during pain that is becoming chronic [30]. Using this paradigm in patients with FM—one subgroup with and one without irritable bowel syndrome (IBS)—it was shown that FM patients without IBS sensitized in the habituation learning condition. FM patients with IBS demonstrated neither learning of sensitization nor habituation. Thus, operant perceptual learning seems to be impaired in patients with FM [31].

Another type of learning that is important for pain modulation is Pavlovian conditioning, where originally neutral stimuli become associated with pain and can later by themselves enhance pain perception and induce chronicity. In a typical aversive Pavlovian differential delay conditioning procedure, aversive pictures were paired with painful electric stimulation, whereas positive pictures were paired with the absence of shock [32]. CBP patients showed an enhanced muscular response of the left forearm (where the unconditioned stimulus (US) was applied) to the reinforced conditioned stimulus (CS) already in the pre-conditioning phase, indicative of more anticipatory anxiety towards the painful stimulus. During learning, the painful muscle showed an increased response to the reinforced conditioned stimulus and an increased response to the reinforced and unreinforced conditioned stimulus in the extinction phase. The contingent negative variation of the EEG differentiated between the conditioned stimulus and the unconditioned stimulus in healthy controls, but not in the CBP patients. These results are indicative of an altered anticipatory brain response in CBP patients. In a similar paradigm using visual signs and thermal stimuli, only 50% of the patients with FM compared to 100% of the HCs were aware of the US–CS contingency [33]. The CS had only significant effects on the heart rate in the HCs and the aware FM subjects, but not in the unaware FM subjects, suggesting that deficits in contingency learning may increase anxiety and, consequently, pain sensation.

## 3. Therapy

### 3.1. Pharmacotherapy

The number of drugs evaluated for the treatment of FM has constantly and substantially increased over the last decade. The recently published European League against Rheumatism (EULAR) revised recommendations for the management of fibromyalgia evaluated ten substances (substance classes) with respect to pain as key outcome parameter (but also including fatigue, sleep, and daily functioning). Based on the Grading of Recommendations Assessment, Development and Evaluation system [34], (weak) recommendations were put forward for amitriptyline, pregabalin/gabapentin, cyclobenzaprine, duloxetine/milnacipran, and tramadol [35] (Table 1). Among them, only three drugs have been approved by the U.S. Food and Drug Administration for the treatment of pain in FM: one substance that binds to the α_2_δ subunit of a voltage-dependent presynaptic calcium channel (pregabalin) and two selective serotonin (5-HT) and norepinephrine (NE) reuptake inhibitors (duloxetine and milnacipran). However, the best-studied drug for the treatment of FM is amitriptyline—a non-selective 5-HT and NE reuptake inhibitor that has been shown to be efficacious in numerous studies [36,37], and which many authors consider to be the first-line drug in the pharmacological treatment of pain in FM [38]. Amitriptyline also has a beneficial effect on fatigue and poor sleep. Pharmacologically, it is a strong modulator of the NE transporter and a moderate modulator of the 5-HT transporter, blocking the reuptake of NE and/or 5-HT and subsequently increasing their intrasynaptic concentrations. Selective serotonin reuptake inhibitors were found to be less efficacious [39,40] than the tricyclic compounds, suggesting an important role of NE for the analgesic effect. Two selective serotonin and norepinephrine reuptake inhibitors—duloxetine and milnacipran (MLN)—have undergone recent multicenter trials, and were shown to be efficacious in a number of outcome variables in FM, such as self-reported pain, stiffness, number of tender points, physical functioning, and fatigue [41,42]. Antiepileptic drugs are also widely used for the treatment of various chronic pain conditions, including postherpetic neuralgia and painful diabetic neuropathy [43]. Pregabalin and gabapentin have both been shown to reduce pain and sleep disturbances in FM patients, but had no effect on depressed mood [44,45]. Both substances bind to the α_2_δ subunit of a presynaptic calcium channel and reduce the calcium influx at nerve terminals, causing a decrease in the release of excitatory neurotransmitters such as glutamate and substance P. For more complete reviews on pharmacological treatment options, see [4,46,47].

**Figure 1:**
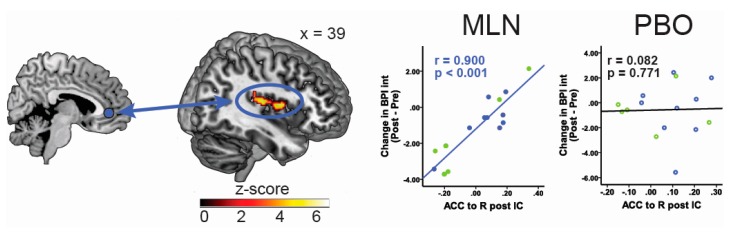
Pre-treatment resting state functional connectivity predicts decrease in pain interference in response to MLN treatment. Displays pre-milnacipran (MLN) treatment connectivity as a predictor for pain response to MLN. Results displayed contain seed-to-target connectivity (seed regions displayed on left) and plots of significant regressions for the MLN treatment arm and corresponding statistics for the placebo treatment period. ACC = anterior cingulate cortex, BPI Int = Brief Pain Inventory interference scores, IC = insular cortex, IPL = inferior parietal lobule, L = left, MLN = milnacipran, PBO = placebo, R = right.

A number of studies have shown that pharmacological interventions can modulate brain response to painful stimuli, such as an increase in pressure pain induced neural activation in the posterior cingulate cortex following MLN intake [48]. As previously indicated, current research suggests that aberrant functional connectivity between pain-processing brain regions may underlie the pathogenesis of FM and other chronic pain states, and is potentially a better biomarker of clinical pain. As such, rs-fc can also be viewed as a promising parameter to monitor changes in brain function associated with pharmacological treatment and/or predict treatment response. In a recently-published study, our group was able to show that decreased functional connectivity between pro-nociceptive regions and anti-nociceptive pain regions at baseline (before treatment), specifically between the rostral part of the anterior cingulate cortex and the IC, as well as between the periaqueductal gray and the IC were associated with reductions in clinical pain scores during MLN therapy; i.e., patients with lower preexisting functional connectivity had the greatest reduction in clinical pain [49]. Figure 1 illustrates the association between rs-fc and pain response to MLN. Furthermore, Harris et al. [50] demonstrated that the treatment of FM patients with pregabalin leads to a reduction in insular glutamate/glutamine concentrations associated with a dissociations in rs-fc between the IC and the inferior parietal lobule (a key structure within the default mode network), which was in turn correlated with reduction in clinical pain [50].

### 3.2. Behavioral Interventions

The assumption that chronic pain is greatly influenced by learning and memory processes suggests that treatment should focus on the alteration of these memory traces. Behavioral and cognitive methods or their combination are especially well-suited for this purpose because they can specifically modulate alterations in brain function or brain chemistry present in a specific pain condition, whereas pharmacological treatments act in a more unspecific manner. The operant behavioral training specifically aims at high levels of pain behaviors. The goals of this training are: (1) to decrease pain behaviors in an effort to extinguish pain; (2) to increase activity levels and healthy behaviors related to work, leisure time, and the family; medication reduction and management; and (3) to change the behavior of significant others [25]. The overall goal is to reduce disability by reducing pain and increasing healthy behaviors. To avoid negative reinforcement learning, medication is switched from a pain-contingent to a fixed time schedule, where medication is given at certain times of the day. The enhancement of activity and the reduction of inactivity and invalidity will be targeted with similar principles. Studies have shown the effectiveness of this training in patients with FM as well as other pain syndromes such as CBP [51,52], and it is especially effective in reducing pain behaviors. After an operant behavioral treatment in FM, a shift from an emotional motivational processing of experimental pain to a more sensory discriminative processing was reported [53]. There was a close correlation of the effect of the training and the brain response for the experimental pain stimuli.

The cognitive-behavioral model of chronic pain emphasizes the role of cognitive, affective, and behavioral factors in the development and maintenance of chronic pain [54]. The cognitive-behavioral training modifies pain-eliciting and maintaining behaviors, cognitions, and emotions to reduce feelings of helplessness and lack of control with the aim of establishing a sense of control over pain. Therefore, patients are taught several techniques to deal with pain episodes as cognitive restructuring, pain coping strategies, and relaxation and imagery techniques. Cognitive-behavioral pain management has been shown to be a very effective treatment of chronic pain [55]. Whereas operant treatment especially reduces pain behaviors and also pain intensity, cognitive-behavioral therapy has a special effect on the affective and cognitive aspects of pain [52]. It was suggested that a cognitive-behavioral treatment changes the brain’s processing of pain through an altered cerebral loop between pain signals, emotions, and cognitions, which leads to an increased access to executive regions for reappraisal of pain [56]. High catastrophizing thoughts—which were correlated with an increased resting state functional connectivity between the primary somatosensory cortex and the anterior IC—could be reduced by cognitive-behavioral therapy and accompany a reduced resting state connectivity between those regions [57]. Since extinction is more difficult than acquisition, principles of extinction training need to be considered [58]. For an overview of randomized controlled trials using operant- or cognitive-behavioral treatments, see [59]; for a discussion of the potential psychobiological mechanisms, see [60].

## 4. Conclusions and Outlook

The goal of this review was to provide an overview of some aspects of the pathophysiology of FM, with a focus on brain imaging and resting state functional connectivity on the one hand, and psychological concepts on the other hand. However, it should be noted that despite the advances that have recently been made in our understanding of this condition, a unifying concept of FM is still lacking, and it is likely that diverse mechanisms contribute differently to the clinical picture in different individuals.

We also tried to provide an overview of some treatment options in FM—both pharmacological and non-pharmacological (specifically behavioral interventions). Although the pharmacological substances used in FM are well characterized with respect to their mechanisms of action and chemical binding sites, the exact location in the CNS where they exert their analgesic effects remains to be fully elicited. Interestingly, at least some drugs (i.e., amitriptyline and pregabalin) seem to modulate large-scale networks within the brain, rather than acting only on the spinal level (i.e., affecting nociceptive transmission within the dorsal horn), which is the mechanism most frequently put forth when discussing their analgesic action. Importantly, the effect of any single drug examined in groups of individuals is modest, just like analgesics tested in other chronic pain states. This situation emphasizes the need for combined pharmacotherapies and the incorporation of pharmacotherapy into a broader program of non-pharmacological therapies.

In this regard, treatments that combine pharmacological interventions with behavioral and cognitive-behavioral interventions are an important approach that in the future needs to be investigated more systematically [61]. In anxiety disorders, for example, it has been shown that exposure with or without additional pharmacological intervention can alter brain processes related to stimuli that are relevant for the disorder. It has been found that pharmacological agents such as d-cycloserine—a partial NMDA (N-Methyl-D-Aspartat) receptor antagonist—can be effective in enhancing the extinction of aversive memories. In several exposure studies, d-cycloserine has been used as an effective adjunction [62,63]. Another promising agent is cannabinoid, which can effectively modulate extinction [64,65] and therefore might be further investigated in extinction trainings. Since pain seems to generally increase excitability, substances that decrease excitation (e.g., gabapentin or pregabalin) would also seem suited to serve as enhancers of extinction. To counteract the context specificity of extinction training, as many environments and behaviors as possible should be included. For the prevention of relapses, a training with episodes of stress and pain is important. In addition, cognitive and emotional aspects of pain need to be targeted [58].

In FM, there are only a few studies that have investigated the combination of both pharmacological and psychological therapies, which in everyday practice hold promise to further improve pain and concomitant symptoms in FM, as compared to an unimodal approach. Apart from beneficial effects on a behavioral level (e.g., reduced pain behaviors or an increase in pharmacological compliance mediated by non-pharmacological treatment), such multi-component approaches might also unfold synergistic effects on the neural level. This should be investigated in more depth in future studies. It will also be of outstanding importance to further subdivide FM patients with respect to their co-morbidities (e.g., FM with and without IBS, FM with and without anxiety disorders, etc.). Co-morbidities are likely to be important indicators of subtle differences in the underlying pathophysiology which require different pharmacological approaches and/or a different emphasis on either pharmacological or non-pharmacological (e.g., psychological) interventions.

## Figures and Tables

**Table 1:** Substances for which a recommendation (for the treatment of fibromyalgia) is put forward by the European League against Rheumatism (EULAR).

Name	Substance Class	Mechanism of Action	Recommended Dosage	Grade of Recommendation *
**Amitriptyline**	Tricyclic antidepressant	Inhibition of the presynaptic serotonin and norepinephrine transporter; 5-HT_2A_, 5-HT_2C_, 5-HT_6_, 5-HT_7_ receptor antagonism	10–50 mg/day	weak for
**Cyclobenzaprine**	Muscle relaxant, tricyclic antidepressant derivative	Inhibition of the presynaptic serotonin and norepinephrine transporter; 5-HT_2A_ receptor antagonism	10–40 mg/day	weak for
**Duloxetine**	Antidepressant, serotonin and norepinephrine reuptake inhibitor	Selective serotonin and norepinephrine reuptake inhibition	20–120 mg/day	weak for
**Milnacipran**	Antidepressant, serotonin and norepinephrine reuptake inhibitor	Selective serotonin and norepinephrine reuptake inhibition	100–200 mg/day	weak for
**Pregabalin**	Anticonvulsant	Modulation of the α_2_δ subunit of a presynaptic calcium channel	300–450 mg/day	weak for
**Gabapentin**	Anticonvulsant	Modulation of the α_2_δ subunit of a presynaptic calcium channel; increased GABA turn over	1200 mg/day	weak for
**Tramadol**	Weak opioid	Weak μ-receptor agonism, norepinephrine reuptake inhibition	150 mg/day	weak for

* Grading of Recommendations Assessment, Development and Evaluation system for making recommendations [34]. 5-HT = serotonin. GABA = γ-aminobutyric acid.

## Abbreviations

5-HT	Serotonin
DMN	Default mode network
FM	Fibromyalgia
GABA	γ-Amino-butyric-acid
IC	Insular cortex
MLN	Milnacipran
fMRI	Functional magnetic resonance imaging
NE	Norepinephrine
rs-fc	Resting state functional connectivity
US	Unconditioned stimulus
CS	Conditioned stimulus
CBP	Chronic back pain
HCs	Healthy controls
IBS	Irritable bowel syndrome
EULAR	European League against Rheumatism
IPL	Inferior parietal lobule
ACC	Anterior cingulate cortex
BPI Int	Brief Pain Inventory interference scores
NMDA	N-Methyl-D-Aspartat
